# Self-Expandable Metal Stent (SEMS) Versus Lumen-Apposing Metal Stent (LAMS) for Drainage of Pancreatic Fluid Collections: A Randomized Clinical Trial

**DOI:** 10.7759/cureus.37731

**Published:** 2023-04-17

**Authors:** Marcos Eduardo Lera dos Santos, Igor Mendonça Proença, Diogo Turiani Hourneaux de Moura, Igor Braga Ribeiro, Sergio Eiji Matuguma, Spencer Cheng, João Remi de Freitas Júnior, Gustavo de Oliveira Luz, Thomas R McCarty, José Jukemura, Eduardo Guimarães Hourneaux de Moura

**Affiliations:** 1 Serviço de Endoscopia Gastrointestinal do Departamento de Gastroenterologia, Hospital das Clínicas da Faculdade de Medicina da Universidade de São Paulo (HCFMUSP), São Paulo, BRA; 2 Serviço de Endoscopia Gastrointestinal do Departamento de Gastroenterologia, Hospital das Clínicas da Faculdade de Medicina da Universidade de São Paulo (HCFMUSP), Sao Paulo, BRA; 3 Division of Gastroenterology, Hepatology and Endoscopy, Brigham and Women’s Hospital, Harvard Medical School, Boston, USA; 4 Division of Gastrointestinal Surgery, Hospital das Clínicas da Faculdade de Medicina da Universidade de São Paulo (HCFMUSP), Sao Paulo, BRA

**Keywords:** lams, sems, lumen-apposing metal stent, self-expandable metal stent, pancreatic pseudocyst, encapsulated pancreatic collection, pancreatic fluid collection, eus-drainage

## Abstract

Background and aim

Endoscopic ultrasound (EUS)-guided drainage is the gold standard approach for the treatment of encapsulated pancreatic collections (EPCs) including pseudocyst and walled-off pancreatic necrosis (WON), and is associated with an equivalent clinical efficacy to surgical drainage with fewer complications and less morbidity. Drainage may be achieved via several types of stents including a fully covered self-expandable metallic stent (SEMS) and lumen-apposing metal stent (LAMS). However, to date there have been no randomized trials to compare these devices. This study aimed to compare the efficacy and safety of the SEMS versus LAMS for EUS-guided drainage of EPCs.

Methods

A phase IIB randomized trial was designed to compare the SEMS versus LAMS for the treatment of EPCs. Technical success, clinical success, adverse events (AEs), and procedure time were evaluated. A sample size of 42 patients was determined.

Results

There was no difference between the two groups in technical (LAMS 80.95% vs 100% SEMS, p=0.107), clinical (LAMS 85.71% vs 95.24% SEMS, p=0.606) or radiological success (LAMS 92.86% vs 83.33% SEMS, p=0.613). There was no difference in AEs including stent migration rate and mortality. The procedure time was longer in the LAMS group (mean time 43.81 min versus 24.43 min, p=0.001). There was also a difference in the number of intra-procedure complications (5 LAMS vs 0 SEMS, p=0.048).

Conclusion

SEMS and LAMS have similar technical, clinical, and radiological success as well as AEs. However, SEMS has a shorter procedure time and fewer intra-procedure complications compared to non-electrocautery-enhanced LAMS in this phase IIB randomized controlled trial (RCT). The choice of the type of stent used for EUS drainage of EPCs should consider device availability, costs, and personal and local experience.

## Introduction

Pancreatic and peri-pancreatic fluid collections (PFCs) may result from episodes related to acute or chronic pancreatitis, trauma, or pancreatic surgery. Although most patients that develop PFCs remain asymptomatic with frequent regression of collections with conservative management alone, other sequelae may develop to include infection, refractory abdominal pain, delayed gastric emptying, and compression of the common bile duct. For patients with symptoms or collections that do not respond to conservative management, drainage is indicated [1,2].

The development of therapeutic endoscopic ultrasound (EUS), as well as adequate and dedicated devices, has enabled EUS-guided drainage to become the preferred approach of encapsulated pancreatic collections (EPCs), including pancreatic pseudocysts and walled-off pancreatic necrosis (WON) [3-5]. At present, EUS-guided drainage is associated with a high clinical efficacy - similar to surgical drainage - but associated with fewer adverse events, reduced morbidity and better quality of life after procedure [6]. While EUS-guided treatment remains a highly effective treatment, there is currently no consensus on the best stent to achieve adequate drainage.

At present, several types of stents have been developed, each with different materials, sizes, and stent deployment techniques. One of the first and best-established stents used successfully for this purpose was the double pig-tail plastic stent (DPS). Since that time, use of a fully or partially covered self-expandable metal stent (SEMS) was shown to potentially provide improved drainage due to the stent’s larger diameter [7-11]. Due to the design of conventional SEMS and potential for migration, a more novel lumen-apposing metal stent (LAMS) was developed [12,13].

Despite a multitude of available stents to achieve EUS-guided drainage of PFCs, there has been only one randomized clinical trial (RCT) comparing the use of DPS versus LAMS - finding no difference in clinical success, number of reintervention, or adverse events (AEs) [14]. Additional systematic reviews with meta-analyses comparing plastic versus metallic stents have been published with conflicting results - five studies comparing DPS versus LAMS [15-19] and two studies comparing DPS versus all metal stents [20,21].

Despite this literature, much of which is retrospective in nature, no RCT has effectively compared success and safety of SEMS versus LAMS for the treatment of PFCs. Given the larger diameter of metal stents and frequent preferred adoption over DPS for drainage, this question remains clinically important to endoscopists. Therefore, the primary aim of this study was to compare the efficacy and safety of the SEMS versus LAMS for EUS-guided drainage of EPCs through a phase IIB RCT.

## Materials and methods

Study approval 

This was a phase IIB RCT designed in accordance with the CONSORT 2010 criteria of updated guidelines for reporting randomized parallel groups [22]. This study was approved by the Research Ethics Committee of the University of São Paulo School of Medicine, Approval no. CAAE: 06203418.1.0000.0068 and registered on the “Registro Brasileiro de Ensaios Clínicos (ReBEC)” under the number RBR-4mdb59.

Study design and randomization

This was a 1:1 RCT comparing two stents for endoscopic EUS-guided drainage of EPCs. Patients were enrolled from March 2018 to May 2020, at a single center at the Gastrointestinal Endoscopy Service of the Hospital das Clínicas da Faculdade de Medicina da Universidade de São Paulo (HC-FMUSP), a quaternary service specializing in advanced therapeutic endoscopy and EUS. Randomization was performed using a dedicated software, with 1:1 pairing in blocks of four and six. The generated sequence was placed in opaque sealed envelopes numbered 1 to 42 by a physician who was not participating in the study. After puncturing the collection with a 19G fine needle aspiration (FNA), the envelope corresponding to the case number was opened and the randomization revealed to the endoscopist.

The endoscopist who performed the procedure was not blinded due to the need to perform the appropriate technique and procedure. Patients and researchers who performed the outcome analysis were blinded to the type of stent used. All patients were screened by the lead investigator (blinded) following pre-established eligibility criteria. All patients were blinded to allocation which included use of two stent types: 1) fully covered SEMS (10 mm diameter x 60 mm length) versus 2) non-electrocautery enhanced LAMS (12 mm diameter x 30 mm length). All included patients signed a consent form. 

Participants

Inclusion Criteria

Patients over 18 years old and patients with documented evidence of PFC (documented history of acute or chronic pancreatitis regardless of etiology and diagnosis of pancreatic pseudocyst or WON based on computed tomography (CT), magnetic resonance imaging (MRI) or EUS-guided imaging with clinical indication drainage (infection, abdominal pain, delayed gastric emptying, or compression of the common bile duct).

Exclusion Criteria 

Patients without an adequate window to perform EUS-guided drainage; patients with a history of previous percutaneous drainage; asymptomatic patients or patients with PFC less than four weeks after acute pancreatitis or those with disorganized collection (i.e. no encapsulation); inability to access the collection (ie, surgically altered anatomy not amenable to drainage) or history or recent intracollection hemorrhage (ie, pseudoaneurysm); patients younger than 18 years.

Sample size

As this is a phase IIb RCT with no previous data available in the literature that could be used as a basis to perform a reliable sample calculation, then a sample of 42 patients was determined (21 per group).

Procedure and technique

All procedures were performed under general anesthesia using a linear echoendoscope and fluoroscopic assistance. EUS was performed immediately prior to drainage with assessment of the following parameters: collection location, best drainage window, collection size, wall-to-collection distance, content aspect (with or without debris) and vascular structures. If the patient was not currently receiving antibiotic therapy, a dose of antibiotic was administered prior to drainage (ciprofloxacin 400 mg and metronidazole 500 mg IV) and a seven-day course post-procedure.

**Figure 1:**
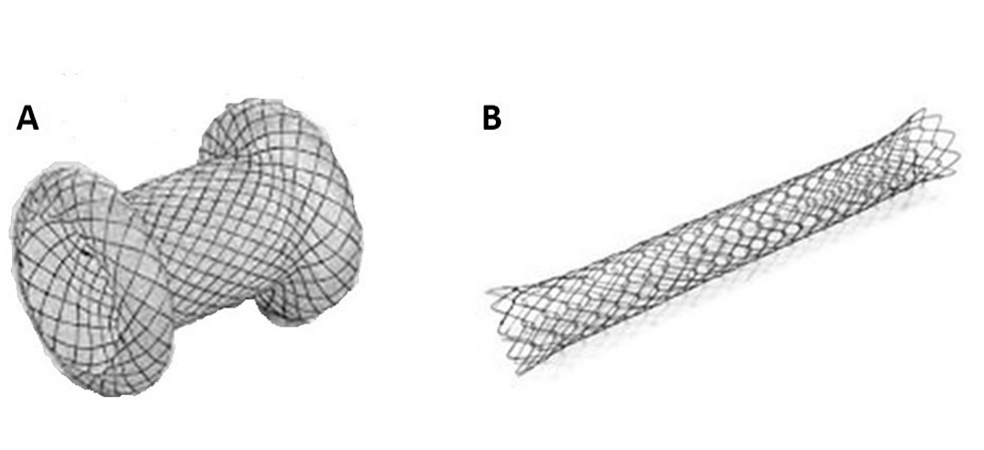
Metal stents A: Lumen-Apposing Metal Stent (LAMS)                                                      B: Self-Expandable Metal Stent (SEMS)

After initial EUS evaluation, puncture of the PFC was performed with a 19G FNA. Fluid was aspirated and sent for analysis (amylase, carbohydrate antigen 19-9 [CA 19-9], carcinoembryonic antigen [CEA]) followed by passage of a 0.035-inch guidewire passage. Once the guidewire was confirmed to be within the PFC, passage of a 6 Fr cystotome was undertaken and the tract dilated with a 4 to 6 mm hydrostatic balloon. This process was identical for both groups (SEMS and LAMS) with type of stent then selected for randomization prior to deployment. In this study, the LAMS was standardized to the Plumber Hanarostent (Figure 1A) with the SEMS standardized to fully covered Hanarostent biliary metal stent (M.I.Tech, Seoul, South Korea) (Figure 1B).

Post-procedure monitoring

Endoscopic pancreatography (Wirsungraphy) was scheduled for all patients between one and two weeks after the initial procedure with passage of a pancreatic stent if any leak, stricture or duct disruption was identified within the main pancreatic duct (MPD). Patients with unsatisfactory clinical or laboratory evolution were evaluated for additional procedure: wash with saline and hydrogen peroxide, direct endoscopic necrosectomy (DEN) and/or percutaneous drainage by interventional radiology.

All patients were followed for a period of 12 months. Interval CT was performed at one, three, six and 12 months after the initial drainage. The maximum dwell metal stent time, regardless of the group, was pre-determined at four weeks. Patients with resolution of the collection on the one-month via CT and without MPD disruption underwent metal stent cystgastrostomy removal. Patients without complete resolution of the collection on the one-month via CT and/or patients with MPD disruption underwent removal of the metal stent with exchange for a DPS.

Outcome measures

The main outcomes for this study were clinical success and safety. Other outcomes included technical success, radiological success, procedure time, intra-procedure complications and AEs related to EUS-guided drainage of the PFC. Technical success was defined as ability to achieve successful stent placement, defined as distal flange of stent within the PFC and proximal flange within the lumen of the stomach or duodenum, without any salvage maneuver. Clinical success was defined by resolution of patient- or collection-associated symptoms with radiologic success defined by complete collapse of collection or residual collection (measured as <2 cm on major diameter). Adverse events monitoring included episodes of bleeding (early, within seven days of cystgastrostomy versus late, developed after seven days), perforation, and stent migration. AEs were stratified according to the 2010 American Society for Gastrointestinal Endoscopy (ASGE) lexicon [23]. Procedure- or collection-associated mortality was also collected and analyzed. 

**Figure 2:**
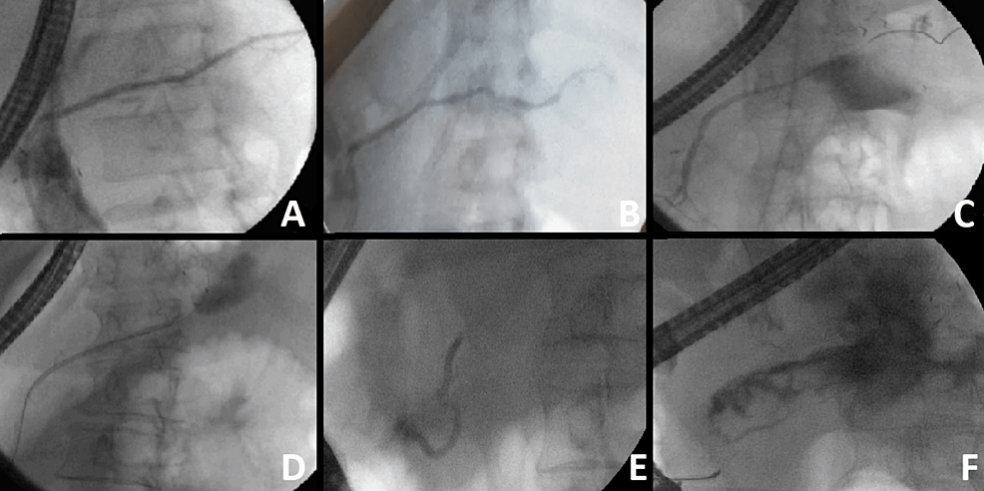
Lera-Proença Classification for Wirsungraphy (A) normal pancreatography (type I); (B) stricture (type II); (C) partial disruption (type III); (D) total disruption with contrast extravasation (type IV-A); (E) total disruption without contrast extravasation and cut-off (Type IV-B); (F) stricture and complete disruption with contrast extravasation (Type II + IV-A)

Additional measured variables of interest included intra-procedure complications (analyzed separated from AEs) - which includes any problem during stent correct placement, PFC recurrence, procedure duration, and findings during endoscopic pancreatography. Procedure time was measured from time to FNA puncture to stent deployment. Endoscopic pancreatography findings were documented according to the Lera-Proença classification (Figure 2) [24]. 

Statistical analysis

Baseline patient and PFC characteristics as well as measured outcomes were summarized as means ± standard deviation for continuous data and frequencies and proportions for categorical data. Continuous data were compared using the two-sample t-test or Wilcoxon rank-sum test and categorical data were compared using the Chi-square or Fisher’s exact test, as appropriate. Statistical significance with two-tailed alpha level of 0.05 was defined as a two-tailed P value <0.05. For all analyses, data were analyzed in the intention-to-treat population.

## Results

Recruitment and patient characteristics

**Figure 3:**
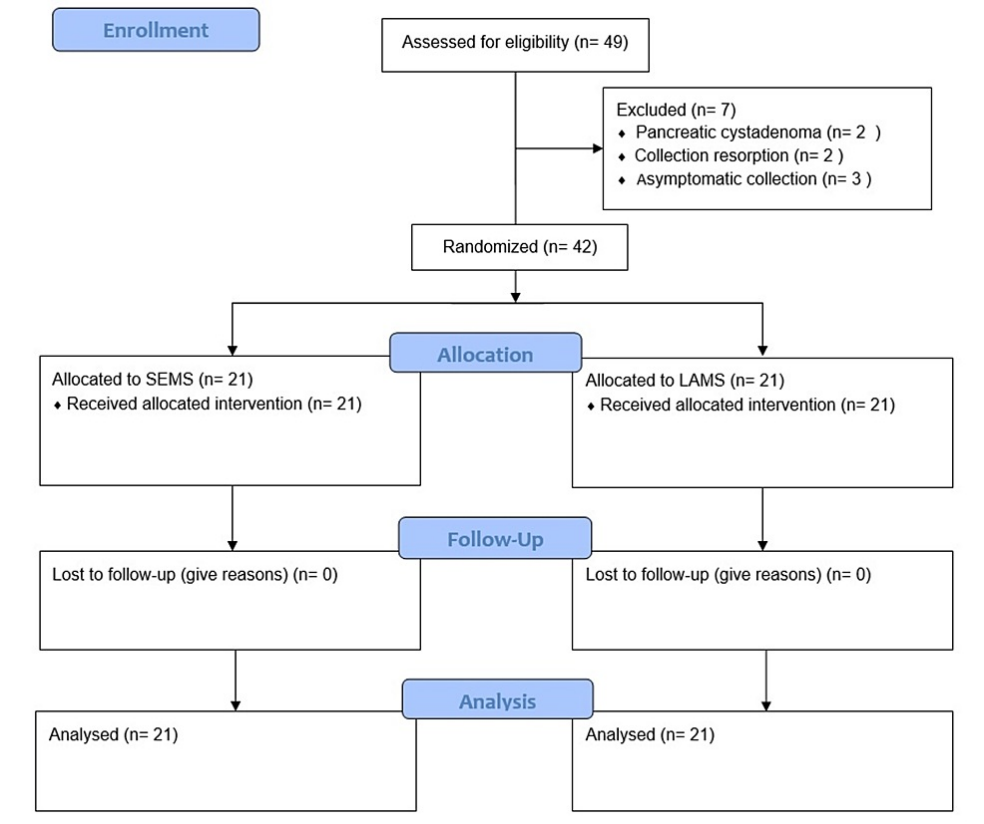
CONSORT flow diagram SEMS: self-expandable metallic stent LAMS: lumen-apposing metal stent

From March 2018 to May 2020, a total of 49 patients were evaluated. Seven patients were excluded - two for spontaneously PFC reabsorption, three patients were asymptomatic, and two patients had imaging suggestive of a cystadenoma (confirmed after FNA analysis). Therefore, a final total of 42 patients were randomized, 21 for each study group (Figure 3).

All patients completed at least 12 months of follow-up. Among the included patients, 57.14% were male with a majority of patients (59.52%) having developed WON. The main etiology was biliary pancreatitis (47.62%) with a mean collection size of 12.94 cm in the LAMS group and 13.34 cm in the SEMS group (p>0.05). There were no differences between groups in any baseline characteristics (Table 1, 2).

**Table 1:** Baseline characteristics of patients and collections LAMS: Lumen-apposing metal stent SEMS: Self-expandable metal stent WON: Walled-off pancreatic necrosis

	Group				Total (n = 42)		p-value
	LAMS (n = 21)		SEMS (n = 21)				
	n	%	n	%	n	%	
Gender							
Female	9	42.86	9	42.86	18	42.86	1.000
Male	12	57.14	12	57.14	24	57.14	
Collection type							
WON	13	61.90	12	57.14	25	59.52	1.000
Pseudocyst	8	38.10	9	42.86	17	40.48	
Etiology							
Alcohol	9	42.86	10	47.62	19	45.24	1.000
Biliary	10	47.62	10	47.62	20	47.62	
Idiopathic	2	9.52	1	4.76	3	7.14	

**Table 2:** Baseline characteristics of patients, collections, and cystic fluid analysis. LAMS: Lumen-apposing metal stent SEMS: Self-expandable metal stent SD: Standard deviation CEA: Carcinoembryonic antigen CA: 19-9: Carbohydrate antigen 19-9

	Group				
	LAMS		SEMS		p-value
	Mean (±SD)	Median (P25 – P75)	Mean (±SD)	Median (P25 – P75)	
Age	45.05 (±3.36)	42.00 (32.00 – 55.0)	49.29 (±2.95)	51.00 (39.00 – 61.50)	0.349
Collection size	12.94 (±1.15)	10.10 (8.70 – 17.65)	13.34 (±0.70)	14.00 (11.15 – 15.05)	0.365
CEA	26.29 (±14.52)	3.56 (0.89 – 13.54)	6.80 (±1.60)	2.59 (1.60 – 9.79)	0.780
CA 19-9	10.989 (±4542)	431.10 (285 – 22430)	4.060 (±2291.95)	578 (17 – 4561)	0.297
Amylase	16.293 (±8.529)	3.887 (900 – 17.560)	2.6231 (±7.670)	8588 (1.172 – 39.909)	0.307

Procedure characteristics

There was a difference in the number of intra-procedure complications, five in the LAMS group and zero in the SEMS group (p=0.048). Of the five complications, four were related to errors in releasing the flanges, but all were successfully corrected after rescue technique. There was one bleeding during the procedure, which was controlled after complete deployment of the LAMS. Transgastric access was the most commonly used approach in both groups, with only one transduodenal (duodenal bulb) drainage in each group (Table 3).

**Table 3:** Characteristics of EUS-guided drainage procedures LAMS: Lumen-apposing metal stent SEMS: Self-expandable metal stent EUS: Endoscopic ultrasound

	Group				Total (n = 42)		p-value
	LAMS (n = 21)		SEMS (n = 21)				
	n	%	n	%	n	%	
Puncture site							
Transgastric	20	95.24	20	95.24	40	95.24	1.000
Transbulbar	1	4.76	1	4.76	2	4.76	
Intra-procedure complication							
Yes	5	23.81	0	0.00	5	11.90	0.048
No	16	76.19	21	100.00	37	88.10	

Despite these similarities, procedure time was longer in the LAMS group, with a mean time of 43.81 min (SD +- 6.55 min) compared to 24.43 min (SD +- 1.99 min) (p=0.001) of the SEMS group.

Technical, clinical, and radiological success

The technical, clinical, and radiological success was 80.95%, 85.71%, and 92.86% in the LAMS versus 100%, 95.24%, and 83.33% in the SEMS group, respectively (all p>0.05). Radiological success after six months was 92.86% in the LAMS versus 83.33% in the SEMS group (p=0.613).

Adverse events and mortality

There was no significant difference between groups, with one AE occurred in the LAMS group (fatal late bleeding) with a total of four AEs in the SEMS group (one stent migration, one mild early bleeding, one mild late bleeding, and one moderate late bleeding) (Table 4).

**Table 4:** Outcomes LAMS: Lumen-apposing metal stent SEMS: Self-expandable metal stent

	Group					Total		p-value
	LAMS			SEMS				
	n	%	n		%	n	%	
Migration								
Yes	0	0.00	1		5.00	1	2.78	1.000
No	17	100.00	19		95.00	36	100.00	
Related deaths Non-related deaths	3 3	50.00	1 1		50.00	4 4	50.00 50.00	1.000
Adverse Events								
Migration	0	0.00	1		25.00	1	16.67	1.000
Early bleeding	0	0.00	1		25.00	1	16.67	
Late bleeding	1	50.00	2		50.00	3	50.00	
Recurrence								
Symptomatic	1	50.00	0		0.00	1	20.00	0.400
Asymptomatic	1	50.00	3		100.00	4	80.00	
Technical success								
Yes	17	80.95	21		100.00	38	90.48	0.107
No	4	19.05	0		0.00	4	9.52	
Clinical success								
Yes	18	85.71	20		95.24	38	90.48	0.606
No	3	14.29	1		4.76	4	9.52	
Radiological success (up to 6 months)								
Yes	14	77.78	16		80.00	30	78.95	1.000
No	4	22.22	4		20.00	8	21.05	
Radiological success (6 to 12 months)								
Yes	13	92.86	15		83.33	28	87.50	0.613
No	1	7.14	3		16.67	4	12.50	

There were three deaths related to the EPC in the LAMS group and one in the SEMS group, but this difference was not significant. Three deaths were due to sepsis (one in SEMS and two in LAMS group) and one due to late bleeding (LAMS group).

Additional procedures and recurrences 

Thirty-eight of the 42 patients (90.48%) underwent Wirsungraphy by endoscopic retrograde cholangiopancreatography (ERCP) to evaluate the MPD. Two patients died prior to Wirsungraphy and two additional patients underwent the procedure, however, it was not possible to cannulate the MPD (i.e., procedural failure). Among the 38 patients with Wirsungraphy, we identified four (10.5%) Wirsungraphy type I (normal), one (2.6%) type II (MPD stricture), five (13.1%) type III (MPD partial disruption), 18 (47 .4%) type IV-A (complete MPD disruption with contrast extravasation), seven (18.4%) type IV-B (complete MPD disruption without contrast extravasation) and three mixed (Table 5).

**Table 5:** Wirsungraph type according to Lera-Proença classification and recurrence

Wirsungraphy type	N (%)	Recurrence
I	4 (10.5%)	0
II	1 (2.6%)	0
III	5 (13.1%)	0
IV-A	18 (47.,4%)	3
IV-B	7 (18.4%)	1
II+III	1 (2.6%)	0
II+IV-A	1 (2.6%)	0
III+IV-A	1 (2.6%)	0

There were five episodes of PFC recurrence, two in the LAMS group and three in the SEMS group (p=0.400). Of the five recurrences, four occurred in patients with type IV Wirsungraphy, three in type IV-A and one in type IV-B. The other recurrence occurred in a patient with unsuccessful MPD cannulation and is shown in Tables 3, 4. One patient (2.6%) developed mild acute pancreatitis after Wirsungraphy with resolution after two days with conservative treatment.

Of the 42 patients that underwent EUS-guided endoscopic drainage, two (8.3%) required additional endoscopic-guided drainage procedures (multi-gate approach), both in the SEMS group. Four patients (9.5%) required adjunctive drainage therapies including percutaneous drainage by interventional radiology, two from each group. Three of these patients (75%) achieved clinical success after combined drainage, while one progressed to death in the LAMS group. A total of 12 patients (28.5%) underwent DEN, five in the LAMS and seven in the SEMS group. Twenty-four patients (57.1%), 12 in each group, underwent collection wash with saline and hydrogen peroxide.

## Discussion

This is the first RCT to compare SEMS versus LAMS for EUS-guided transmural drainage for EPCs. Since the development of LAMS, many centers have adopted this treatment strategy as a first-line modality for the drainage of EPCs, especially in cases of WON. Even prior to widespread adoption of LAMS, a pivotal randomized trial by the Dutch Pancreatitis Study Group established endoscopic transgastric drainage as superior to traditional surgical debridement for the treatment of walled-off necrosis [25]. This endoscopic approach is less invasive compared to a traditional surgical approach and allows for drainage of one or multiple collections [26] and improved access to the collection in case of washing or DEN [27]. 

Based on the results of this study, there was no difference between the LAMS versus SEMS with regard to technical, clinical, and radiological success. Although there is no statistically significant difference in terms of procedure success, one study suggested both strategies are superior to DPS which have been shown to be associated with lower clinical success rates, higher adverse events, and increased procedure times [28]. However, the Dutch Pancreatitis Study Group recently published a study showing similar outcomes between DPS vs LAMS for EUS-drainage of WON [29], raising the debate again. It should be noted that in our study SEMS had 100% technical success while LAMS had 80.95%, though this was not significant. Furthermore, there were more intra-procedure complications in the LAMS group compared to the SEMS group. 

These findings may be explained by the fact that this specific LAMS delivery system used in our study is more technically complex and allows a much smaller margin for errors during its stent deployment, especially in the release of the proximal flange, since it is 3 cm long and the flanges must be well adjusted to the gastric and collection walls. On the other hand, the SEMS is 6 cm long with no need to be precisely adjusted, which made it easier to deploy. Furthermore, due to the intricacies of the LAMS delivery system used, the learning curve may have influenced these results where 75% of LAMS issues occurred in the first half of the trial. It is also important to note that although the endoscopists who performed the procedure have experience with the use of LAMS, they did not have experience with this model of deployment system.

Despite some cases of stent deployment with the LAMS, all were resolved and drainage of the PFC effectively achieved - either grasping the LAMS with cold forceps or deploying another stent through the LAMS - a DPS or a SEMS, as demonstrated in a previous publication [30,31]. Additionally, the type of LAMS is also important to acknowledge, as this stent type is not coupled with an electrocautery system, likely resulting in a longer average release time with LAMS compared to SEMS. With the development of novel LAMS with a “hot” electrocautery-enhanced system, the procedure time is extremely short, since most of the steps and devices used in the classical technique are no longer required [32-34].

Importantly, there was also no difference in AEs between the two stent types. The only available RCT comparing LAMS versus DPS demonstrated a large number of late adverse events in the LAMS group, reaching an unacceptable rate of 32.3% of adverse events in an interim analysis. Almost all of these AEs were observed five weeks after drainage and bleeding was the most frequent and severe AE, with stent-related delayed bleeding due to collapse of the cavity adjacent to the metallic stent and erosion into retroperitoneal vessels [14]. As such, LAMS and SEMS were removed after a maximum of four weeks per our protocol, contributing to the lower rates of AEs in both groups compared to previous literature. 

Although LAMS was designed to avoid stent migration - with two flanges and a shorter length to “appose” tight collection and gastrointestinal wall - our study did not find a statistically significant difference between the two groups. Again, this may be a result of the short four-week dwell time of both stents. Additionally, the absence of difference may be due to a small sample size to assess this outcome. Though the LAMS should have a theoretical advantage compared to SEMS in design to reduce migration, larger powered studies may better assess if differences in migration exist between the two types of stents.

Based on our experience, Wirsungraphy classification helps to standardize the assessment of the MPD and to guide possible approaches [24]. In the included population, we observed a large number of cases with complete MPD disjunction (type IV), which represented almost 66% of the Wirsungraphys performed. Interestingly, all cases of recurrence occurred in patients with Lera-Proença type IV Wirsungraphy, associated with disconnected pancreatic duct syndrome (DPDS), an entity often under-diagnosed and related to a worse prognosis and relapse [27,34]. It is important to highlight that when DPDS is suspected or confirmed a transmural indwelling DPS should be placed and left for a long period to avoid recurrence. 

One issue related to metallic stent drainage in patients with DPDS is the optimal time to remove the metallic stent and replace it with a DPS. One study reported a 23.1% DPS replacement failure three to four weeks after initial drainage with LAMS in patients with DPDS, with a recurrence rate of 21.4% in this subgroup of patients [27]. In our study, we performed Wirsungraphy and replaced the metallic stent for DPS when indicated one to two weeks after initial drainage, with no failure of DPS placement. Despite this data, the optimal time to replace the metallic stent without complete collapse of the collection remains unanswered and should be evaluated on an individual patient basis. 

Interestingly, one well-designed RCT comparing replacing metallic stent by DPS (stent group) four weeks after EUS-drainage in patients diagnosed with DPDS versus no replacing metallic stent (no-stent group) showed no difference in PFC recurrence between the two groups on three months (stent group three vs three no-stent group) and 12 months follow-up (stent group seven vs 13 no-stent group) [35]. Despite the final result, the authors themselves recognized that one of the possible reasons that could have influenced results was the long time (four weeks) to replace the metallic stent, with 11.5% failure to deploy DPS. Besides, there were some cases in which the DPS was hanging in the gastric lumen anchored only by the pigtail - which should not provide adequate drainage. As Rana et al. argued in a letter about this RCT, besides the time after drainage to deploy DPS, other reasons should be considered, such as the amount of functional pancreatic parenchyma left upstream of the disconnection that influences the possibility of PFC recurrence [36]. DPDS is still an underdiagnosed and challenging condition, many aspects regarding endoscopic management after EUS-drainage are still unanswered, such as the perfect time to replace metal stent to DPS, the clinical impact and safety of long-term indwelling DPS and when to remove or not the indwelling DPS [37].

Despite being the first RCT comparing SEMS versus LAMS in the management of EPCs, our study is not without limitations. This study possessed a limited sample size, and thus, the absence of statistically significant differences in some outcomes may be related to being underpowered. Although the endoscopists who performed the procedure have experience with the use of LAMS, they did not have experience with this model of deployment system. Additionally, use of a non-cautery-enhanced LAMS may make this study more difficult to generalize among centers where cautery-enhanced LAMS delivery is widely available. Ultimately, determination of an optimal stent for EUS-drainage of EPCs will continue to evolve with considerations including individual endoscopist experiences, costs, and familiarity. Additional studies are needed to better identify which patients may benefit from a specific type of stent, preferably based on RCTs which are lacking in the literature. 

## Conclusions

The LAMS and SEMS perform effectively for the EUS-guided drainage of EPCs. The SEMS and the non-electrocautery-enhanced LAMS were associated with similar technical success, clinical success, radiological success, as well as safety profile for EUS-drainage of EPCs. However, SEMS was associated with a shorter procedure time and fewer intra-procedure complications in this phase IIB RCT, using non-electrocautery LAMS.
